# When should we expect microbial phenotypic traits to predict microbial abundances?

**DOI:** 10.3389/fmicb.2012.00268

**Published:** 2012-08-02

**Authors:** Jeremy W. Fox

**Affiliations:** Department of Biological Sciences, University of CalgaryCalgary, AB, Canada

**Keywords:** trait-abundance correlations, coexistence, competitive exclusion, local adaptation, frequency dependence

## Abstract

Species' phenotypic traits may predict their relative abundances. Intuitively, this is because locally abundant species have traits making them well-adapted to local abiotic and biotic conditions, while locally rare species are not as well-adapted. But this intuition may not be valid. If competing species vary in how well-adapted they are to local conditions, why doesn't the best-adapted species simply exclude the others entirely? But conversely, if species exhibit niche differences that allow them to coexist, then by definition there is no single best adapted species. Rather, demographic rates depend on species' relative abundances, so that phenotypic traits conferring high adaptedness do not necessarily confer high abundance. I illustrate these points using a simple theoretical model incorporating adjustable levels of “adaptedness” and “niche differences.” Even very small niche differences can weaken or even reverse the expected correlation between adaptive traits and abundance. Conversely, adaptive traits confer high abundance when niche differences are very strong. Future work should be directed toward understanding the link between phenotypic traits and frequency-dependence of demographic rates.

## Species' traits as predictors of their abundances

The underlying causal processes that determine organismal distribution and abundance presumably reflect phenotypic traits rather than taxonomic identity. It is the phenotypic traits of an organism, not its taxonomic identity, that actually determine its rates of reproduction, mortality, and movement (these rates will of course also depend on current and past biotic and abiotic environmental conditions). For this reason, ecologists working on both microorganisms and macroorganisms have called for a turn away from a focus on taxonomic identity and toward a focus on traits (e.g., Lavorel and Garnier, 2002; McGill et al., 2006; Violle et al., 2007; Green et al., 2008). A focus on traits might be particularly useful in microbial ecology. Microbial “species” are difficult to define, but microbial “functional traits”—phenotypic traits that directly or indirectly determine reproduction and mortality—are increasingly easy to measure (e.g., Fierer et al., 2007; Green et al., 2008; Gudelj et al., 2010; Edwards et al., 2012; Lennon et al., 2012).

Many studies using traits as predictors of distribution and abundance explicitly or implicitly assume that there is a straightforward and direct causal connection between the measured traits and abundance. Studies of spatial variation in species composition often assume that local environmental conditions “filter out” organisms with inappropriate traits (e.g., Keddy, 1992; Lavorel and Garnier, 2002; Cavender-Bares et al., 2004). On this view, sites are occupied only by organisms with traits sufficiently well-adapted to the local environmental conditions. In microbial systems, extremophiles provide a clear example: a 70°C hot spring will only harbor microbes with traits allowing them to survive and reproduce at 70°C (e.g., traits such as possession of proteins that retain a functional conformation at 70°C). Many less-extreme examples exist (e.g., Fierer et al., 2007; Schwaderer et al., 2011). For instance, relative abundance of freshwater phytoplankton varies with light availability: species with physiological traits conferring high fitness in low-light environments are most abundant in low-light lakes, while species with physiological traits conferring high fitness in high-light environments are most abundant in high-light lakes (Schwaderer et al., 2011). This view of the environment as filtering out (or at least making rare) species with the “wrong” traits resonates with Baas Becking's famous remark concerning microbial distributions: “everything is everywhere, but the environment selects.” On this view, what evolutionary biologists term “local adaptation” is ubiquitous: the organisms found at any given site will tend to be those that are fittest at that site, and taxa will tend to occur at the sites where they are fittest (Kawecki and Ebert, 2004; Hereford, 2009). Similarly, studies of relative abundance within sites often assume that abundance is at least roughly proportional to how well-adapted taxa are to local conditions (e.g., Pärtel et al., 2001; Poullin and Mouillot, 2004; Harpole and Tilman, 2006; Shipley et al., 2006, 2011; Fierer et al., 2007; Partensky and Garczarek, 2010). For instance, Pärtel et al. (2001) attributed the high relative abundance of “core” alvar grassland plant species, as compared to non-core species, to the possession by core species of traits like low stature, conferring “adaptation to low-fertility conditions.” As a microbial example, the cyanobacterium *Prochlorococcus* is relatively abundant compared to other microbes in oligotrophic tropical and subtropical ocean sites. This has been attributed to its relatively small size and reduced genome, as these adaptive traits reduce nutrient requirements, thereby improving relative fitness in nutrient-poor environments (Partensky and Garczarek, 2010).

However, there are both empirical and conceptual reasons to question whether variation in species composition and relative abundance generally should map so neatly onto trait variation. On the empirical side, studies that directly test for local adaptation by reciprocally transplanting organisms among sites typically do find local adaptation—but it is often quite weak (Hereford, 2009). This is true in microbes as well as in macroorganisms (Belotte et al., 2003). Local adaptation is trivially obvious (and so is rarely tested) when different sites have strongly contrasting environments (e.g., a 70°C hot spring vs. a 20°C pond). But when differences among localities are less extreme, local adaptation can become not just non-obvious, but non-existent. One important reason for this, though not the only one, is dispersal among sites, which decouples local abundance from local demography (Kisdi, 2002; Kawecki and Ebert, 2004; Paul et al., 2011).

However, I will focus on a conceptual problem with species' traits as predictors of their abundances, relevant even when local demography completely determines local abundances. Consider the expectation that the species best adapted to local environmental conditions will tend to be the most locally abundant. This expectation raises a question: why doesn't the best-adapted species simply exclude all the others? The intuitive answer is that it's generally unrealistic to expect competition to lead to exclusion of all but the best-adapted species. Rather, interspecific niche differences will prevent weaker competitors from being excluded. But if that's the case, why expect species' traits to predict their abundances at all, even roughly? Insofar as there are niche differences—species with different traits “make their living” in different ways and so compete less strongly with heterospecifics than conspecifics—then there is no single best-adapted species. Rather, species' demographic rates will depend on their relative abundances (frequencies), with rare species having an advantage over common ones because common species necessarily experience mostly intraspecific competition while rare species necessarily experience mostly interspecific competition (Chesson, 2000). Laboratory experiments with microbes provide many of the most rigorous demonstrations of niche differences leading to stable coexistence (Grover, 1997; Rainey and Travisano, 1998; Le Gac et al., 2012). But to my knowledge the consequences of niche differences for trait-abundance correlations have not been much explored in either micro- or macroorganisms (but see Harpole and Suding, 2007).

Here I use a simple, classic theoretical model to ask how niche differences affect trait-abundance correlations. Under what circumstances can species with traits conferring high adaptedness coexist with, but also maintain higher abundance than, species with traits conferring lower adaptedness? I focus on trait-abundance relationships within a single site for the sake of simplicity, but the results also have implications for variation in species' relative and absolute abundances across sites. This simple model is not a realistic description of any particular natural system, microbial or otherwise, but it is not intended to be. Rather, the model is intended to sharpen intuition and aid hypothesis development. It is widely assumed that locally abundant species should have traits making them well-adapted to local environmental conditions (e.g., Pärtel et al., 2001; Poullin and Mouillot, 2004; Harpole and Tilman, 2006; Shipley et al., 2006, 2011; Harpole and Suding, 2007; Violle et al., 2007; Partensky and Garczarek, 2010). If this intuitively appealing hypothesis is valid, then it ought to hold in the context of a deliberately simplified theoretical model lacking complicating factors like dispersal. And if the hypothesis is not valid, the model can help identify the reasons why not, thereby aiding development of better hypotheses.

## A simple model of trait-abundance correlations

The model is that of MacArthur (1970). The model considers two or more consumer species competing for two or more nutritionally substitutable limiting resources such as different sugars. Consumer *j* consumes resource *i* at a constant per-capita rate *c*_*ij*_, converts a consumed unit of resource *i* into *b*_*ij*_ new consumer individuals, and dies at constant per-capita rate *m*_*j*_. Resource *i* grows logistically with intrinsic rate of increase *r*_*i*_ and carrying capacity *K*_*i*_. Logistic growth is more appropriate for living, self-reproducing resources than non-living resources like carbon compounds, but other forms of resource growth, such as chemostat-type resource supply, would not alter the results. Assuming a well-mixed system in which reproduction and mortality occur continuously, consumer and resource dynamics are given by

(1a,b) $$\begin{array}{l} \frac{dR_{i}}{dt}=r_{i}R_{i}\left(1-\frac{R_{i}}{K_{i}}\right)-\sum_{j}c_{ij}R_{i}C_{j}\\ \frac{dC_{j}}{dt}=\sum_{i}c_{ij}b_{ij}R_{i}C_{j}-m_{j}C_{j} \end{array}.$$

The behavior of this model is well-studied, particularly for certain analytically tractable special cases (reviewed in Chesson, 1990). This facilitates interpretation of the results presented below. To use the model to study trait-abundance correlations, I considered model parameters as consumer “traits.” As noted above, we expect species' phenotypic traits to predict their abundances because those traits affect species' demographic rates. In the context of this model, the *c*_*ij*_, *b*_*ij*_, and *m*_*j*_ parameters are features of the biology of consumer *j* which determine its demographic rates as a function of resource levels. These parameters, therefore, can be considered as consumer traits.

In general, it is not possible to solve analytically for equilibrium consumer abundances as a function of the model parameters, so I relied on numerical simulations. In order to aid interpretation, I considered a special case in which there is a clear-cut separation between traits governing how well-adapted different consumer species are to their shared environment, and traits governing the amount of niche differentiation among different species. I varied “adaptedness” by allowing per-capita mortality rates to vary among consumer species. The lower the *m*_*j*_ value, the better consumer species *j* is adapted to the habitat. I vary niche overlap by varying the *c*_*ij*_ values, while constraining all consumer species *j* to have the same sum of their *c*_*ij*_ values (i.e., ∑_*i*_*c*_*ij*_ = *T* for all *j*). Consumers thus varied in the relative rates at which they consumed different resources, but no consumer was intrinsically better than any other at consuming resources in general. I further assumed that all *b*_*ij*_ = *b*, so no consumer was better than any other at converting consumed resources into new consumers, and no resource was more valuable or nutritious than any other. I assumed that all *r*_*i*_ = *r* and all *K*_*i*_ = *K*, so all resources grew in an identical fashion, independent of consumers. Thus, *m*_*j*_ values completely dictated the intrinsic “adaptedness” of consumers to the shared environment.

Classical analyses of this model measure consumer niche differentiation by making very strong assumptions about the distributions of the *c*_*ij*_ values (Roughgarden, 1989). These strong assumptions are made for mathematical convenience and aren't necessary here. Instead, I follow Chesson (1990) and measure niche differentiation in a more generally applicable way: as “linear independence” of the *c*_*ij*_ values. Imagine plotting the *c*_*ij*_ values of one consumer against those of another. If the points lie on a straight line through the origin, then the two consumers have completely non-independent diets: the *c*_*ij*_ values of one consumer are identical to, or differ by only a constant of proportionality from, the *c*_*ij*_ values of the other consumer. Such consumers exhibit no niche differentiation and cannot coexist at equilibrium (Chesson, 1990). Conversely, if the two consumers do not consume any of the same resources, then their *c*_*ij*_ values are linearly independent. They exhibit maximally large niche differentiation, do not compete at all, and so are guaranteed to coexist as long as each would be capable of persisting on its own. I used equation (12) from Chesson (1990) to measure linear independence between each pair of consumers, and used the mean linear independence of all possible pairs as a measure of the average or overall strength of niche differentiation for the community as a whole. Mean linear independence equals 0 when all consumers have identical diets, and 1 when no consumer shares a resource with any other consumer.

I simulated communities of 10 consumers and 10 resources, with *m*_*j*_ values chosen randomly from a uniform distribution and *c*_*ij*_ values chosen randomly under various constraints so as to explore the full range of possible values of mean linear independence. I ran each simulation to equilibrium, and calculated the Spearman rank correlation between consumer equilibrium abundances and *m*_*j*_ values. I calculated mortality-abundance correlations for all 10 species, and for only persisting species (those with non-zero equilibrium abundances). There were no resource extinctions in most simulations. My choices of initial consumer and resource species richness, parameter values, and initial conditions were arbitrary, but the results are robust to these arbitrary choices.

## Model results

**Figure 1:**
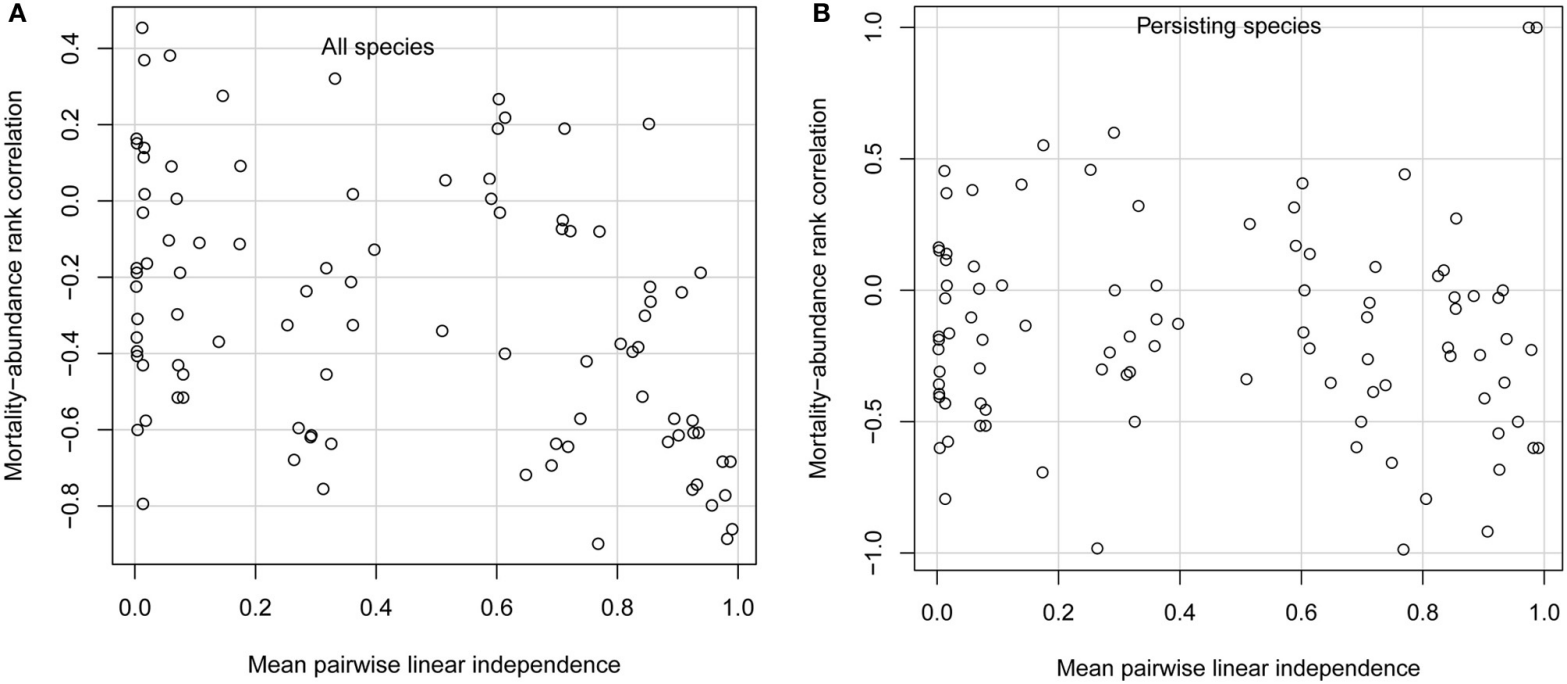
**Simulation model results.** Spearman rank correlation between consumer per-capita mortality rate and consumer equilibrium abundance, for **(A)** all 10 consumer species, and **(B)** only persisting consumer species, as a function of mean linear independence of consumer diets. Each open circle gives results from one simulation. Note that, in the limiting case when every species consumes a different resource, so that species' diets are completely independent of one another, the rank correlation would be −1 (not shown).

When species had very similar diets, only those few species with the lowest per-capita mortality rates persisted. This was due to competitive exclusion; for the parameter values I used, each species would persist if it were growing on its own. When niche differences were weak, the only important difference among species was their mortality rates. In this case, only species with the lowest mortality rates, and thus the highest adaptedness, can persist (Chesson, 1990; Grover, 1997). In the limit when all species have identical diets, the single species with the lowest mortality rate competitively excludes all others. Competitive exclusion of all but the few species with the lowest mortality rates leads to a strongly negative mortality-abundance correlation across all species (Figure 1A; points in extreme lower right of panel). However, considering only the few persisting species, the mortality-abundance correlation can take on any value (Figure 1B).

Species with low mortality rates also attain higher abundances when niche differences are as large as possible, meaning that every species consumes a different resource so that interspecific competition is absent. That is, when species have such large niche differences that they don't interact at all, their abundances are determined solely by their adaptedness to the habitat. In this limiting case, the rank correlation between adaptedness and abundance is −1 (not shown in Figure 1). Thus, both extremely small and extremely large niche differences create conditions favoring high abundance of species with traits conferring high adaptedness.

But in between these extremes, a wide range of trait-abundance correlations can arise. Whether niche differences are small (but not so small as to lead to exclusion of most species), large (but not so large as to prevent any interspecific competition), or intermediate, mortality-abundance correlations can range from strongly negative to moderately positive (Figures 1A,B). Positive mortality-abundance correlations indicate that less well-adapted species with higher per-capita mortality rates attain higher abundance. For non-extreme levels of niche differentiation, mortality-abundance correlations are highly variable when considering all species, or only persisting species. This means that species with low per-capita mortality rates are almost as likely to be competitively excluded as species with high per-capita mortality rates. On average, the typical mortality-abundance correlation is moderately negative, but the variability around this average is much the most striking feature of the results.

This variability in the strength and direction of the trait-abundance correlation arises because, when there is any diet overlap at all, species compete. Further, they don't just compete with others sharing the same resources, but interact indirectly with every species in the community (e.g., species 1 and 3 interact indirectly if both share resources with species 2). This means that a species' equilibrium abundance depends not just on its own traits, but depends in a complex way on the traits of every species in the community. Even quite small diet overlap can decouple equilibrium abundance from adaptedness, because even quite small diet overlap causes all species to interact with one another.

The same results hold if the trait governing adaptedness is not mortality rate, but some other trait such as total per-capita feeding rate or efficiency at converting consumed resources into new consumers (results not shown). Traits conferring high adaptedness to local conditions reliably confer high abundance only when niche differences are either maximally large or nonexistent. Non-extreme levels of niche differentiation, even if quite small, frequently weaken and even reverse expected trait-abundance correlations.

## Discussion

In many respects, the deliberately simplified scenario considered here is a best-case scenario for trait-abundance correlations. The ecology of the system (Equation 1) is known, and is very simple. For instance, there are no environmental fluctuations or perturbations that might cause species' abundances to temporarily deviate from those expected based on their trait values. The system lacks many factors, such as dispersal, known to weaken or alter trait-abundance correlations. A single trait governs adaptedness to the local environment. Trait values and species' abundances, including those of species which are absent due to competitive exclusion, are known without error. Despite all this, trait values often fail to explain species' abundances, or do so only when competitively excluded species are included in the analysis.

Unfortunately, these results don't suggest any “rules of thumb” for when non-extreme levels of niche differentiation will or will not prevent trait-abundance correlations. When levels of niche differentiation are non-extreme, as they usually are, species' realized abundances, and thus trait-abundance correlations, will be sensitive to idiosyncratic details (here, the precise *c*_*ij*_ values).

These results caution against inferring adaptiveness of traits from species' abundances. In general, the most abundant species at a given site will not necessarily possess traits making them better-adapted to local conditions than rare or absent species.

These theoretical results accord with empirical data. Studies of trait-abundance correlations in both micro- and macroorganisms often identify traits that explain a statistically significant but biologically modest fraction of interspecific variation in abundance (e.g., Harpole and Tilman, 2006; Fierer et al., 2007; Schwaderer et al., 2011). Investigators often attribute unexplained variation in abundance to unmeasured traits, dispersal, or other confounding factors. In contrast, the results shown here suggest that biologically modest trait-abundance correlations are likely to be the rule rather than the exception, and so demand no special explanation.

These results have implications for variation in species' absolute and relative abundances across sites. For instance, the results show that extinct species are not always those most poorly adapted to local conditions. This implies that the local environment cannot be viewed as simply “filtering out” the worst-adapted species. Variation in species composition across sites, therefore, cannot necessarily be ascribed to different local environments “filtering out” different species.

An interesting direction for future research would be to try to link measurements of species' traits not just to adaptedness of different species, but also to the strength of niche differentiation. I further suggest that, in empirical studies, niche differentiation is best quantified by manipulating relative abundances and directly measuring the strength of the resulting negative frequency dependence of species' demographic rates. Such direct, trait-independent measurements of the strength of niche differentiation can then form the basis of follow-up studies to identify their phenotypic basis. Quantifying niche differentiation in this way is standard in microbial laboratory experiments (e.g., Rainey and Travisano, 1998; Brockhurst et al., 2006; Zhang et al., 2009; Le Gac et al., 2012), increasingly common in studies of macroorganisms (Harpole and Suding, 2007), and requires no *a priori* assumptions about how species' traits relate to the strength of negative frequency dependence. This approach has already had success with both macro- and microorganisms. For instance, Angert et al. (2009) showed how an interspecific trade-off between traits conferring high growth capacity and traits conferring tolerance of low resource levels combines with fluctuations in rainfall to generate negative frequency dependence and promote coexistence of desert annuals. In microbes, Brockhurst et al. (2006) and Zhang et al. (2009) demonstrated surprisingly strong negative frequency dependence among strains of *P. fluorescens* lacking any obvious trait differences, illustrating the value of testing for niche differentiation directly.

### Conflict of interest statement

The author declares that the research was conducted in the absence of any commercial or financial relationships that could be construed as a potential conflict of interest.
